# All you need to know about equipment validation for sterility testing

**DOI:** 10.1128/jcm.01477-24

**Published:** 2025-08-11

**Authors:** James E. T. Gebo, Kayla M. Feehely, Chase E. Lattimore, Dylan P. Mogavero, Anna F. Lau

**Affiliations:** 1 Sterility Testing Service, Department of Laboratory Medicine, Clinical Center, National Institutes of Health 2511, Bethesda, Maryland, USA; Vanderbilt University Medical Center, Nashville, Tennessee, USA

**Keywords:** cGMP, equipment, sterility testing, cellular therapy, validation, qualification, IOPQ

## Abstract

Product sterility testing requests are becoming increasingly common in clinical microbiology laboratories due to the rapid development of advanced therapies. Most laboratory directors are hesitant to bring on such tests because they fall under current Good Manufacturing Practices (cGMP) regulated by the United States Food and Drug Administration (FDA), where expectations differ from clinical requirements (regulated by the Centers for Medicare and Medicaid Services). When considerations are made for cGMP testing in the clinical lab, most focus is placed on analytical test validation. In this mini-review, we provide an overview of one critical element within the cGMP quality system—validation of equipment, software, and systems through installation, operational, and performance qualification (IQ, OQ, and PQ or IOPQ). This terminology is not common in clinical laboratories, and the IQ and OQ portions are often overlooked, not performed, and/or not documented, although phase II CAP requirements exist (COM.30550 and COM.30575). This mini-review will provide an overview of the IOPQ framework, what is included and how it differs from CAP requirements, important considerations for an IOPQ, and a summary of FDA citations relating to equipment validation. We provide examples using blood culture systems, controlled temperature units (CTUs; e.g., incubator), and the laboratory information management system, given their likelihood for use in cGMP activities in the clinical lab. Importantly, the IOPQ requirements summarized here would have also been relevant to laboratory-developed tests (LDTs) classified as “devices” prior to the March 31, 2025, annulment of the ruling.

## INTRODUCTION

Cellular-, gene-, and immuno-therapies have emerged rapidly over the last decade, offering treatment for a variety of disease states. The first chimeric antigen receptor T-cell (CAR-T cell) product was approved by the United States Food and Drug Administration (FDA) in 2017. Today, the list of FDA-approved cell and gene therapy products has expanded to 44 (1). Globally, as of March 2024, almost 2,700 unique cell therapies are under evaluation in interventional cancer cell therapy clinical trials (2) (ClinicalTrials.gov). In the United States, biological products fall under two categories: (i) minimally manipulated, autologous, Section 361 products; and (ii) more than minimally manipulated, allogeneic, Section 351 products (see Reference [3] for further discussion). Section 351 products are subject to current Good Manufacturing Practices (cGMP) for manufacturing and quality control release testing. The regulatory landscape for Section 361 products and whether they are subject to the cGMP regulations is less clear (see Reference [3] for further discussion).

**Fig 1:**
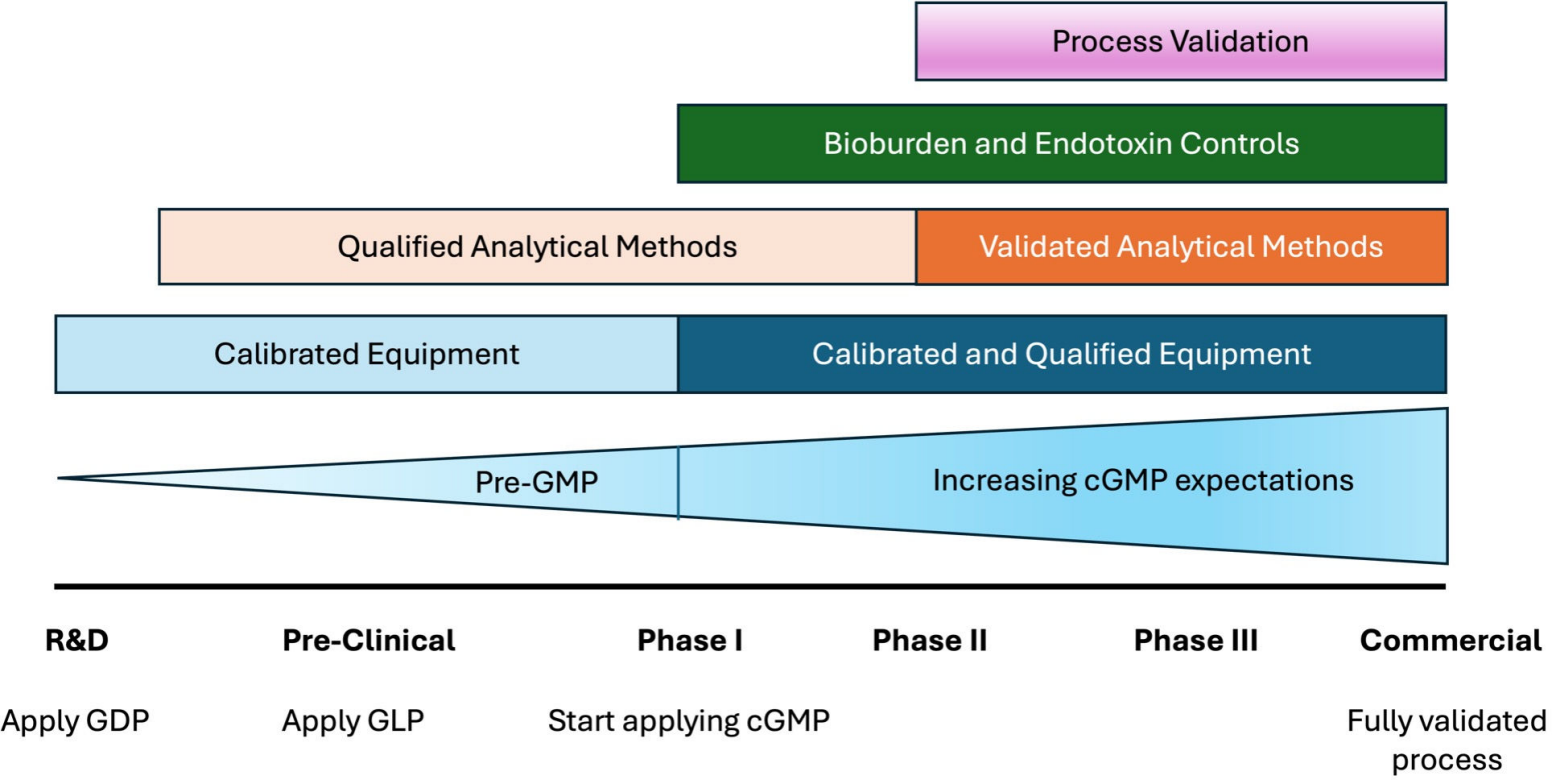
Development of cGMP requirements during the life cycle of the product. Copyright PDA, Inc. (from PDA Technical Report 56 [7]), reproduced with permission. GDP, Good Documentation Practices; GLP, Good Laboratory Practices.

Repeated questions posed on clinical microbiology listservs (ClinMicroNet and DivC) hosted by the American Society for Microbiology (ASM) highlight increasing administrative pressure for clinical microbiology labs to bring on product sterility testing to assist cell therapy labs because in-house testing is less expensive, turnaround time for results is quicker, and on-site microbiology expertise and instrumentation (such as blood culture systems and incubators) are available. Product sterility testing, however, is not a standalone test and may be accompanied by requests to perform other cGMP tests, including environmental monitoring cultures, personnel monitoring cultures (e.g., gloved fingertip testing), aseptic processing simulation cultures (or media fills), and qualification studies. Furthermore, regulatory oversight differs substantially between clinical and cGMP testing laboratories, with the former falling under the Clinical Laboratories Improvement Amendments (CLIA) Act regulated by the Center for Medicare and Medicaid Services (CMS) and the latter falling under the Food, Drug, and Cosmetic Act (FD&C Act) regulated by the FDA (Table 1). CLIA and FD&C Act-regulated fields have different and unique requirements for quality systems and laboratory practices, and it may not be feasible to expect clinical laboratories to meet FDA requirements for cGMP testing, as discussed elsewhere (4, 5). Briefly, the FDA expects that the sterility testing laboratory employs facilities and controls that are not inferior to those used for aseptic manufacturing (6). Additionally, the implementation of cGMP is expected for Phase I clinical trials and is expected to increase as the product matures toward commercialization (Fig. 1) (7).

**TABLE 1:** High-level overview comparing regulatory oversight for laboratories under CLIA and the FD&C

	Human specimens	Food, drugs, and devices
Act	Title 42, part 493. Clinical Laboratory Improvement Amendments of 1988 (CLIA)	Title 21 parts 11, 211, and 820. Federal Food, Drug, and Cosmetic Act (FD&C Act)
Scope	Laboratories performing waived tests and tests of moderate and/or high complexity.	Pharmaceutical products, medical devices (includes IVDs), biotechnology products, food and beverage, dietary supplements, and cosmetics manufactured in the United States or sold to the United States
Regulatory and Law Enforcement Authority	Centers for Medicare and Medicaid Services (CMS)	Food and Drug Administration (FDA)
Standards Development	College of American Pathologists (CAP)	International Organization for Standardization (ISO) for environment classification, United States Pharmacopeia (USP) for test methods.
Inspection frequency	CAP every 2 years, CMS when needed.	FDA based on risk (typically every 3–4 years). More frequent inspections may occur for firms that have a higher risk of non-compliance or for manufacturers of products that require greater scrutiny based on market trends. Inspections may also occur in response to for-cause reasoning (this occurred at the National Institutes of Health in 2015) (8).
Compliance checklists	Provided by CAP	None

Despite these concerns, a recent review showed that 18/23 (78%) clinical microbiology labs have reportedly performed sterility testing for hematopoietic stem cells (3). Additionally, the most recent stem cell processing (SCP) proficiency testing survey (SCP-B 2024) issued by the College of American Pathologists (CAP) showed that 55/59 (93%) participants used BacT/ALERT or BACTEC for sterility testing. The historical lack of FDA oversight for early phase studies and section 361 products suggests that inherited programs may exist in clinical laboratories. Additionally, the rapid growth of the cell and gene therapy field and the lack of sterility guidance from cellular therapy professional societies including the Association for the Advancement of Blood and Biotherapies (AABB), the American Society of Gene and Cell Therapy (ASGCT), the International Society of Cell and Gene Therapy (ISCT), and the Foundation for the Accreditation of Cellular Therapies (FACT) may have contributed to the use of clinical labs for product sterility testing.

A robust quality management system is the backbone of any cGMP program. Fact sheets and guidance documents that summarize how quality systems (through cGMP regulations) are used to establish the foundation for drug product quality have been provided by the FDA (9, 10). This mini-review will focus on one critical element within the cGMP quality system—validation of equipment, software, and systems through installation, operational, and performance qualification (IQ, OQ, and PQ or IOPQ). This mini-review will provide an overview of the IOPQ framework, what is included and how it differs from CAP requirements, important considerations for IOPQ, and a summary of FDA citations relating to equipment validation. We provide examples using blood culture systems, controlled temperature units (CTUs; e.g., refrigerators, freezers, and incubators), and the laboratory information management system (LIMS), given their likelihood for use in cGMP activities in the clinical lab. Importantly, equipment validation differs from equipment calibration. Equipment calibration is a measurement of accuracy compared with a known standard and forms a part of the routine calibration/preventive maintenance program. In contrast, validation is a series of specification tests to ensure that the equipment functions as intended according to its pre-defined specifications.

Briefly, the FDA defines validation as “confirmation by examination and provision of objective evidence that the particular requirements for a specific intended use can be consistently fulfilled” (21 CFR 820.3(z)). This is fulfilled by “…establishing by objective evidence that a process consistently produces a result or product meeting its predetermined specifications.” (21 CFR 820.3(z)(1)). Simply, the FDA expects processes to have sufficient controls to ensure the process can deliver a product/outcome of a desired quality. Process validation, which includes equipment validation, is used to demonstrate that these controls work as intended. The use of calibrated and qualified (i.e., validated) equipment is expected at any level of cGMP application, beginning at Phase I clinical trials (Fig. 1).

Notably, up until March 31, 2025, when the rule was nullified (11), the requirement for equipment IOPQ would have been relevant to approximately 80,000 to 100,000 pre-existing laboratory-developed tests (LDTs) (12, 13) performed across 12,000 registered high-complexity laboratories (5% of US laboratories) (14). This is because the May 6, 2024, ruling classified LDTs as devices and laboratories offering LDTs as device manufacturers, thereby subjecting them to regulations under the FD&C Act (Table 1), needing to meet cGMP quality standards under 21 CFR 820.

## WHAT IS IOPQ AND WHAT ARE THE REGULATORY REQUIREMENTS?

IOPQ is the validation performed on equipment, software, and/or systems used in cGMP processes to ensure that they are installed correctly, operate according to the manufacturer’s specifications, and perform consistently to meet pre-defined acceptance criteria (Table 2). Examples of equipment relevant for clinical laboratories performing cGMP testing may include incubators (including blood culture systems), pipettes, centrifuges, document management systems, and the laboratory information system. The IOPQ follows an approved validation protocol that details specific test scripts and pre-defined acceptance criteria. All areas of the IOPQ are documented, including the validation rationale, test protocols, test results, protocol deviations, corrective actions, and a validation report. These documents are maintained throughout the entire lifespan of the equipment.

**TABLE 2:** Definition of IOPQ for the validation of equipment used in cGMP applications

	Definition
Installation Qualification (IQ)	IQ verifies that the equipment has been received as specified, installed properly, meets the manufacturer’s requirements, and is ready for additional validation work. IQ includes checks on equipment configuration, environmental conditions, and the presence of all required components and documentation. IQ ensures that the equipment is set up correctly in the intended environment and that any necessary utilities (e.g., electrical, plumbing) are correctly connected and meet the minimum requirements.
Operational Qualification (OQ)	OQ tests the equipment’s functionality to ensure it operates as intended under defined conditions. This stage involves running tests that challenge the equipment’s operational parameters to verify that all functions work within the specified limits. OQ is performed after IQ and often includes testing alarms, operational sequences (step-by-step processes), and controls to confirm that the equipment operates reliably and according to the manufacturer’s specifications.
Performance Qualification (PQ)	PQ evaluates the equipment’s performance under real-world conditions to demonstrate that it consistently produces results meeting the predetermined criteria. PQ is performed after successful completion of IQ and OQ and may be included in the same document (typically referred to as IQ/OQ/PQ or IOPQ protocols). The PQ is designed to integrate the written procedure(s), personnel, and materials that will be used under routine processing conditions. Depending on what is being validated, a sufficient number of replicate studies is performed, or the equipment/system is monitored for a sufficient length of time to demonstrate proper performance. Protocols should incorporate “worst case” challenges in addition to testing at the normal/intended operating range. PQ ensures that the equipment, when operated as part of a process, performs reliably and consistently to produce the desired outcome.

In the pharmaceutical industry, vendors may provide services (at an additional charge) to satisfy the IOPQ requirements as outlined in Table 2. Execution of the IOPQ may be performed by the vendor themselves or the vendor may choose to partner with a third party (e.g., BioMerieux Industry outsourced the IOPQ of the BacT/ALERT instrument to an independent group). Smaller vendors, or those without experience in the cGMP space, may not offer IOPQ services. Because of the rigorous validation behind IOPQ and the need for consistency across consumers, templates outlining the specific test scripts needed for IOPQ are often available. Test scripts for IQ and OQ are often standardized and do not vary between laboratories. In contrast, the PQ should be tailored specifically to the use case of the end user. Therefore, PQ scripts form only the framework needed by the end user to design the PQ. A laboratory may choose to perform qualification testing internally using IOPQ templates and test scripts/templates provided by the vendor, although this can be laborious and time-consuming. Importantly, clinical vendors may be unfamiliar with the IOPQ requirements, which are more common in the pharmaceutical industry. Vendors often have different branches of the same company that specialize in the regulatory governance of clinical diagnostics versus cGMP (eg, BioMerieux Industry and Bruker GP are branches that specialize in industrial applications for food, veterinary, and pharmaceuticals under umbrella companies that include clinical portfolios). Given the different regulatory landscape between clinical and cGMP (Table 1), finding the right vendor contact with cGMP IOPQ knowledge is important.

Under the cGMP regulations, equipment validation is mandated through 21 CFR 211.68(a), 820.72, and 820.75, which requires that equipment be suitable for its intended use, capable of being cleaned and maintained to prevent contamination, and does not present a hazard to the quality of the product. The expectations for design and equipment qualification are described further in the FDA Guidance for Industry on Process Validation (section C.1) (15). The FDA and other global authorities require IOPQs to ensure that equipment used in the production and testing of pharmaceutical products and medical devices is reliable and fit for purpose. Electronic systems are also subject to additional IOPQ requirements under 21 CFR Part 11, which spans computer systems to analytical test platforms such as blood culture instruments and real-time PCR instruments. 21 CFR Part 11 expands the scope of IOPQ for electronic systems to include confirmation that electronic records are accurate, complete, and secure through the implementation of controls for software validation, audit trails, data integrity, and system access.

## HOW IS IOPQ DIFFERENT FROM WHAT IS ALREADY REQUIRED BY CAP?

Equipment validation is often an overlooked area in clinical laboratories, although the CAP checklists include language similar to the IOPQ regulatory requirements. The CAP All Common checklist item COM.30550 specifically references “Instrument/Equipment Performance Verification,” stating that “the laboratory verifies the performance of all instruments and equipment prior to initial use, after major maintenance or service, and after relocation…”, requiring records of “appropriate function checks” as evidence of compliance. The definition of “appropriate function checks” is loose and may be interpreted as preventative maintenance and inclusion of quality controls that meet acceptance criteria. The CAP All Common checklist item COM.30575 requires “Instrument/Equipment Operation” and states that “written procedures for start-up, operation, maintenance, and shutdown of instruments and equipment [be available]…” which, in practice, may be interpreted as filing of the manufacturer’s manual. In contrast, the IOQ portion for the validation of equipment used in cGMP activities is more detailed (see examples below). It is probable that, regardless of the two Phase II All Common checklist requirements, the IOQ for equipment in clinical settings may be overlooked or have minimal documentation. Instead, clinical laboratories place most focus on analytical performance qualification (PQ) through assay verification for unmodified FDA-cleared in-vitro diagnostics (IVDs) or assay validation for LDTs (CAP All Common checklist). Importantly, PQ in the clinical field is distinct from PQ applied to equipment for cGMP applications. In the clinical field, analytical test validation (or verification for FDA-approved tests) often involves a test method that has an instrument component (e.g., antimicrobial susceptibility testing platforms, point of care molecular tests), where equipment testing is rolled into the one analytical test validation. Similar instrument-integrated test systems also exist in the cGMP field (e.g., BacT/ALERT and BioFire). However, the PQ requirement in cGMP also pertains to static equipment that may not be directly associated with analytical test interpretation (e.g., a controlled temperature unit, pipette, and biosafety cabinet).

In the following section, we provide three IOPQ case studies for the BacT/ALERT with MYLA software, a controlled temperature unit (e.g., incubator), and the laboratory information management system to help guide clinical labs performing cGMP tests regulated by the FDA.

### Example #1 – blood culture instrument (e.g., BacT/ALERT)

As discussed above, blood culture systems are used frequently in the clinical setting for product sterility testing. Table 3 summarizes the 19 test programs (each consisting of several test scripts) used to execute the IOPQ for the BacT/ALERT with the MYLA software system. The IOPQ is used to verify instrument functionality, MYLA software functionality, and combination functionality. The MYLA software is also evaluated for compliance with 21 CFR Part 11 for electronic records and electronic signatures.

**TABLE 3:** Summary of IOPQ test programs used for BacT/ALERT instrument with MYLA software validation

	Test program	Description
IQ	Hardware and firmware/software verification	Verifies that all required hardware components are available, and all mechanical and software components are properly installed and meet installation specifications and user requirements. Several specifications are checked to verify minimum/expected values or conditions (such as network connectivity, available RAM and hard drive size, processor speeds, available printer, monitor, keyboard, and mouse, indicator lamps on drawers, and that they agitate when closed), and fields are included to record model and serial numbers of components.
	Field verification	Verifies that the system has been installed according to the manufacturer’s recommendations (e.g., sufficient clearance around the instrument, away from direct heat and cold, etc.) and that the unit has been tagged with identification.
	Environmental conditions and utility verification	Verification that the environmental conditions and supplied utilities meet manufacturer recommendations (ambient temperature, relative humidity, AC voltage, connected to a power backup system).
OQ	Temperature optimization and distribution	The temperature of each incubation drawer is monitored over time to determine an average value, which is compared to the setpoint, and the difference is used to determine if temperature adjustment is needed — if the difference is greater than +/-0.5°C, adjustment is required.
	MYLA software user password configuration	Verifies that 21 CFR Part 11 mode is enabled and tests the password protection capabilities of the system (system access requires a valid login, created passwords meet the minimum character requirements, characters are hidden while typing, all username/password combinations are unique, etc.). Testing is completed separately for the BACT/ALERT computer by itself, then in conjunction with the MYLA software because the system may be used with either method.
	BacT/ALERT username and password verification	
	MYLA software user access level and configuration	User accounts have different levels of access for using and managing the system. Testing involves verifying that users can access what they are authorized to perform and that they do not have access to actions they are not authorized to perform. E.g., only Administrator accounts are authorized to create users and passwords and have access to all configuration options and audit trails; Lab Tech accounts should not have access to any of these. Testing is completed separately for the BACT/ALERT computer by itself, then in conjunction with the MYLA software because the system may be used with either method.
	BacT/ALERT user access level and configuration	
	BacT/ALERT control panel/screen verification	The control panel features are each tested, ensuring all available options can be adjusted and that the screen displays the appropriate information in response.
	Instrument cell calibrations	A set of standards is used to calibrate selected cells (i.e., bottle positions) within the chosen drawer. Testing is successful if the cells can be successfully calibrated.
	Enabling and disabling modules, drawers, racks, and cells	Using the Administrator user account, this tests whether incubation modules, drawers, racks, and cells can be successfully disabled and enabled.
	Faults and operator error codes/alarms verification	The most common faults and error codes are triggered to test whether the system alerts users (via audible and visual alarms). Only the most commonly occurring faults will be simulated to avoid causing damage to the equipment. These include conditions such as the incubation module power off, drawer open, controller module power off, cell calibration failure, etc.
	Scanner verification	Operation of the barcode scanner is tested to ensure it accurately reads bottle barcodes. Barcodes transmit sample information such as Bottle Type and Bottle ID#.
	MYLA software and BacT/ALERT operational verification workflow	This tests the operation of the MYLA and BacT/ALERT. The two components must be able to communicate, the BacT/ALERT must alert users when bottles flag positive for growth, accommodate simultaneous sample analysis, and MYLA must be able to generate sample reports.
	Verification of default search/report	Default sample reporting functionality of the BacT/ALERT and MYLA is tested. The BacT/ALERT must capture required sample information, such as sample ID, type, analysis, and time/date, from which MYLA must be able to generate a report. Reports must be accurate and human-readable (i.e., not a list of numbers or software codes).
	BacT/ALERT backup verification	For the BacT/ALERT, manual backup functionality is tested by creating a USB backup of data. For MYLA, backups are set to occur automatically on a routine basis. Backup functionality is tested, ensuring it occurs as scheduled and saves to the correct location.
	MYLA backup verification	
	Bact/ALERT and MYLA audit trail verification	Functionality and accuracy of the audit trail from both the BacT/ALERT and MYLA are verified. To meet acceptance criteria, both must list all user operations, cannot be altered or deleted, each action is listed in chronological order and attributable to a specific time, date, and user, the action and time stamp must be accurate, and the audit trail is human-readable.
PQ	Performance qualification	Assessment of analytical test performance, including specificity, limit of detection, robustness, repeatability, ruggedness, and equivalency as per USP <1223 > (16).

Prior to IOPQ, training of the personnel executing the protocol is verified and documented on relevant vendor documents. All equipment and material information (including calibration window, lot numbers, expiration dates, etc) should be within specification and recorded during IOPQ execution. Calibration certificates are attached to the documentation as evidence of compliance for traceability.

### Example #2 – controlled temperature units (e.g., incubator)

Controlled temperature units (CTUs; e.g., incubators, refrigerators, and freezers) are examples of non-analytical equipment that can directly affect lab testing due to incubation or storage of samples and/or test reagents/materials. In clinical labs, a typical process might include identifying and purchasing a refrigerator that is fit for purpose, installing the refrigerator, choosing a setpoint, placing a calibrated thermometer inside, and allowing the system to equilibrate at the chosen setpoint. Once reached, the CTU refrigerator is considered fit for lab usage.

**Fig 2:**
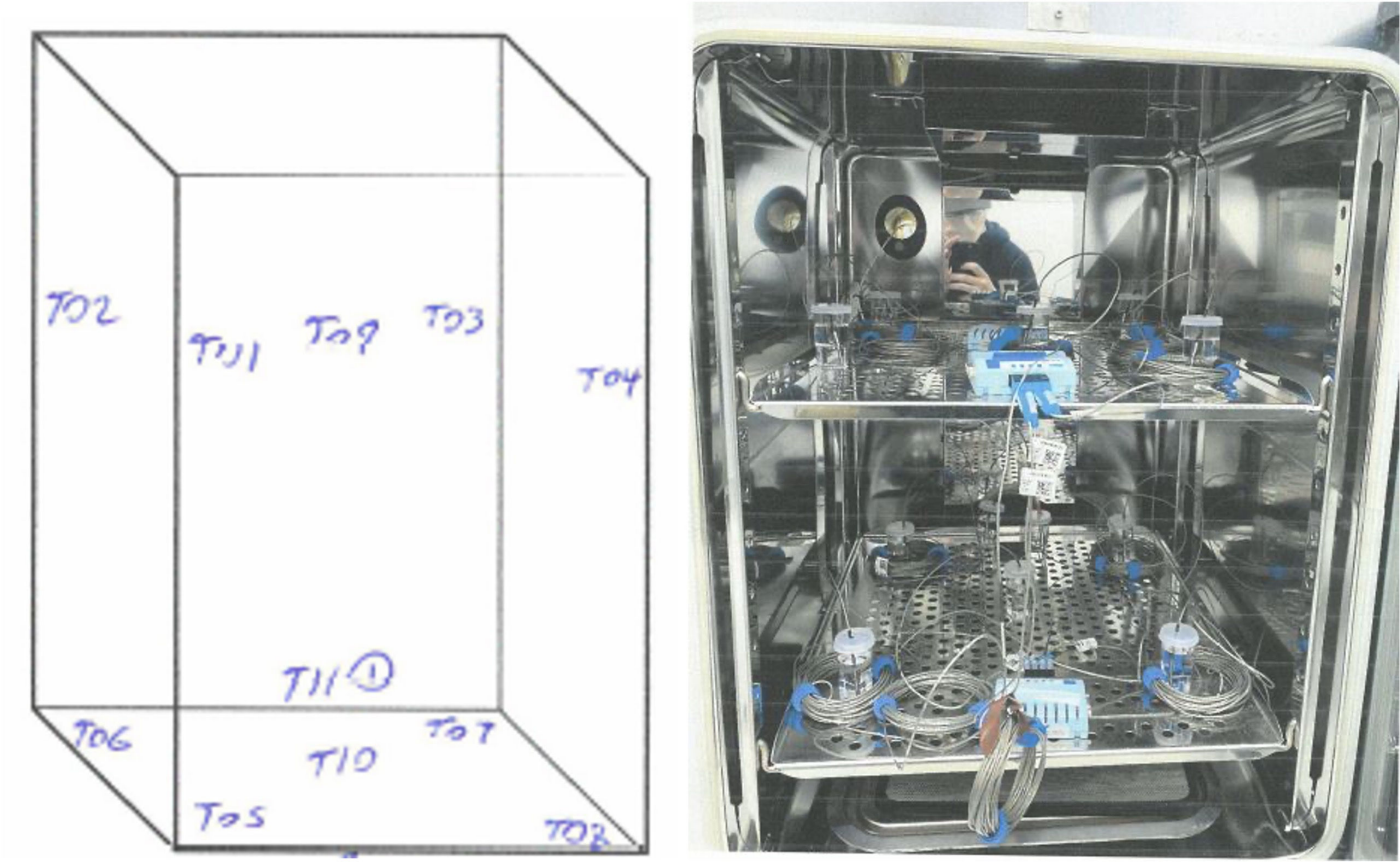
A diagram and a photo illustrating datalogger placement within a CTU for temperature mapping. The datalogger marked with a ① (T11 in the diagram) is placed next to the incubator’s built-in temperature sensor to verify accuracy.

In contrast, the IOPQ of a CTU for cGMP activities is much more complex and requires a considerable volume of documentation. Briefly, IOPQ studies include 24 h temperature mapping (conducted twice—first with an empty chamber then with a filled chamber) to assess for temperature uniformity (Fig. 2), alarm testing, open door recovery studies, and power failure recovery studies (see Table 4 for a summary of the 10 test programs used for IOPQ of a CTU). IOPQ for an incubator (or any other CTU) is performed for a specified temperature setpoint and range to show that the instrument, as installed, is capable of operating at the manufacturer’s design specifications and meeting the end user’s intended usage requirements. IOPQ can be performed for more than one setpoint/range, if desired. An external monitoring system probe is physically located inside the CTU to log temperature in real time and to provide alarm notifications at the chosen alarm setpoints. Importantly, the external monitoring system used for IOPQ of the CTU must be independently qualified prior to use.

**TABLE 4:** IOPQ requirements for a control temperature unit (CTU)

	Test program	Description
IQ	Identifying and documenting SOPs and PM procedures applicable to the operation and maintenance of the incubator	The procedure #, title, version #, and effective date are documented within the IQ protocol, and the SOP is attached. Acceptance criteria are met if the procedure covers operation, maintenance, and calibration of the unit.
	Identifying and verifying that the incubator’s internal temperature sensor and the secondary external monitoring probe have current calibration documentation traceable to NIST.	This includes the temperature sensor built into the incubator as well as the secondary external monitoring temperature probe placed inside the unit. For each, the Instrument Description, identification #, calibration date, and calibration due date are documented within the protocol and certificates are attached. All sensors/probes must be within their calibration window.
	Provide calibration documentation for all instruments/equipment used to perform the IOPQ	This includes a multimeter, a temperature/humidity meter, and all temperature loggers used for the temperature mapping studies in the OQ and PQ. For each, the Instrument Description, serial #, manufacturer/model #, calibration date, and calibration due date are documented, and certificates are attached. All instruments must be within their calibration window.
	Document the manufacturer’s operation and maintenance manual	The Document #, document title, revision and/or date, and location of the physical copy are listed.
	Installation Verification	Verify the manufacturer, model, equipment ID, serial number, temperature setpoint, local high temp alarm, local low temp alarm, leveling (unit is not leaning to one side), incubator location is free from vibration and not in direct sunlight or other sources of heat, sufficient clearance around the incubator, interior dimensions are as expected, door gaskets are free of defects The incubator is part of a Preventative Maintenance/Calibration program, the room conditions (temperature and humidity) meet the specified requirements, the electrical circuit voltage rating and breaker rating meet specifications, verify and record the panel and circuit #s, verify and record the secondary external monitoring temperature probe information (manufacturer, model, ID, serial number)
OQ	Alarms testing	Simulate fault conditions to trigger alarms, then verify that alarms return to normal once fault conditions are removed Low temperature alarm: activates when the setpoint is set above ambient temp, silences when the setpoint is set below ambient temp High temperature alarm: activates when the high temp alarm setpoint is set below ambient temp, silences when the high temp alarm setpoint is set above ambient temp
	Empty Chamber uniformity study	See Fig. 2. Operate the system at the defined setpoint. Define location and # of datalogger temperature probes within the incubator. Program the dataloggers to record the temperature at a specified time interval, leave the unit closed for a minimum of 24 hours, analyze the min/max temperatures achieved and their locations, and compare data with the external monitoring system probe data. Acceptance criteria: all datalogger data generated during the 24-h study period are within the specified temperature range.
PQ	Open door study	This study assesses how long it takes for the unit to go out of the setpoint range when the door is left open. The chamber is empty with a defined # of dataloggers placed at specified locations, including one next to the external monitoring system probe and the RTD (CTU built-in temperature sensor) probe. The data loggers are programmed to collect data at 30-s intervals. Allow the chamber to stabilize at the operating setpoint for at least 30 min, then fully open the incubator door. Leave open for at least 15 min and then close the door. Allow the chamber to stabilize for at least 1 hour, then terminate the study. Analyze the data for the time it took for the first datalogger to read out of range after the door was opened, as well as the time it took all dataloggers to return within range after the door was closed.
	Power failure study	This study assesses how long it takes for the unit to go out of the setpoint range in the event of power loss. The chamber is empty with a defined # of dataloggers placed at specified locations, including one next to the secondary external monitoring probe and RTD probe. The data loggers are programmed to collect data at 30-s intervals. Allow the chamber to stabilize at the operating setpoint for at least 30 min and then remove power. After at least 15 min, restore power. Allow the chamber to stabilize for at least 1 h and then terminate the study. Analyze the data for the time it took for the first datalogger to read out of range after power was cut off, as well as the time it took all dataloggers to return within range once power was restored.
	Loaded Chamber Uniformity	This study demonstrates incubator performance when filled with samples or materials. Operate the system at the defined setpoint. Fill the chamber with incubator packs (e.g., media sleeves in racks) to simulate a loaded chamber. Define location and # of datalogger temperature probes within the incubator. Program the dataloggers to record the temperature at a specified time interval. Leave the unit closed for a minimum of 24 hours, analyze the min/max temperatures achieved and their locations, and compare with the external monitoring system probe data. Acceptance criteria: all datalogger data generated during the 24 hour study period are within the specified temperature range.

### Example #3: electronic data management system (e.g., laboratory information management system)

The laboratory information management system (LIMS) is one of the most complex and integral electronic systems in any laboratory. It acts as the core repository for test data, can automate management of recurring tasks (e.g., calibrations, sample collection, or material reorders), and often is integrated with other laboratory/quality systems (e.g., document management systems and instruments). Because of this level of complexity, a LIMS is typically administered on multiple system environments—development, qualification, and production—to ensure system stability and control of system configurations. As a result, IOPQ of LIMS can be a sizeable endeavor requiring dozens of test programs (Table 5). Importantly, not all test programs are performed in all environments, specifically to prevent validation testing from impacting the production environment or active systems that are connected to LIMS. For example, test programs to assess the correct installation and operation of IT hardware, servers, and software are performed on all environments. In contrast, end-to-end sample lifecycle testing is typically performed in the qualification environment only to avoid generating data in the production environment that will only be used for system testing purposes.

**TABLE 5:** Framework of IOPQ test programs used for LIMS implementation^*a*^

Qualification	System environment	Test program	Description
IQ	Performed on the Development (DEV), Qualification (QUAL), and Production (PROD) environments	Hardware, Software, and Computer Network	Verification that all required hardware (e.g., servers, workstations, switches), software components, and network connections are the correct type/version, installed correctly, and documented.
		Instrument Server(s), if required	Confirmation that instrument server(s) are installed correctly, accessible, and configured as per the system requirements.
		Database Server	Verification that the LIMS database server is installed correctly, meets the required specifications, and is operating as expected.
		Application Server	Verification that the LIMS application server is installed correctly, meets the required specifications, has the required services running, and is operating as expected.
		Core LIMS Configuration	Verification that the base LIMS software is installed, all required core modules are available, and documentation of LIMS version information.
		LIMS Patch Install, if required	Confirmation that any required patches have been installed without error.
		Enterprise Application Platform	Verification that all enterprise platforms (e.g., Java, middleware) are installed, meet system requirements, and documentation of enterprise platform versions.
		Label Management Software	Verification that all native and/or third-party label and barcode software is installed, meets systems requirements, and that installed versions are documented.
		Report Management Software	Confirmation that reporting tools (e.g., Crystal Reports, JasperReports) are installed, meet system requirements, and that the installed versions are documented.
		Statistical Analysis Package Software, if required	Verification that all native and/or third-party statistical software used for data analysis (e.g., SAS, JMP) is installed, meets system requirements, and that the installed versions are documented.
OQ	Performed on the Qualification (QUAL) environment	Verification of IQ Completion and Acceptability	The IQ documentation is reviewed to confirm that all hardware and software components were installed per the user requirements and that no deviations remain open.
		Single Sign-On (LDAP) or Password Policy	Verification that the LIMS user authentication integrates with LDAP or complies with the internal password policy. If confirming compliance with internal password policies, the qualification should include verification that the password character limits meet requirements, lock-out procedures work as expected, and password update notifications/requirements work as expected.
		Core Configuration Changes	Testing of site-specific configuration changes made to the base LIMS to ensure they function as intended. These test scripts typically verify configurations that are defined in the Functional Requirement Specifications to meet pre-defined user requirements.
		Security Roles and Group Configuration	Verification that all required LIMS User Job Types and Data Security settings are configured as per the LIMS Functional Requirement Specification. This test script evaluates whether the job types are appropriately assigned to the correct department(s), that each job type has the required functions assigned to it, and that each job type has access to the required items in the system (e.g., batches, samples, tests, etc.).
		Time Zone Configuration	Verification that LIMS time zone settings are configured correctly and have been applied consistently across time-stamped entries. If the LIMS serves facilities in multiple time zones, the time zone configuration should be verified in each time zone.
		Verification of Active User Accounts	Verify that all active user accounts are set up correctly, have the appropriate permissions/roles, and have the appropriate access levels.
		Verification of Master Data and Calculations	Validation that all master data (e.g., test methods, parameters, consumables, equipment) or calculations that have been imported into the system are accurate and meet user requirements.
		LIMS Simple Connect Instrument Integrations – if required	Verification that all required simple instrument integrations are configured as per the Instrument Functional Requirement Specification. This test script evaluates whether users have access to the required modules in the LIMS to manage simple instruments, each instrument is integrated and configured correctly, and that data can pass between the instrument and LIMS as expected. Simple instruments are those that typically can be connected to a network or printer so that data can be transferred or printed without the need for additional software (e.g., balances, pH meters, etc.). This test program would be performed for each type of instrument or each individual instrument, depending on how your LIMS is set up.
		LIMS Complex Instrument Integrations – if required	Verification that all required complex instrument integrations are configured as per the Instrument Functional Requirement Specification. This test script evaluates whether users have access to the required modules in LIMS to manage complex instrument integrations, that each integration parser is configured correctly, and that instrument data can be correctly parsed to the required LIMS data fields. Complex instruments are those that are typically connected to the network and require additional software to capture/manage assay data. This test program would be performed for each parser/integration or each individual instrument, depending on how your LIMS is set up.
		LIMS Empower Instrument Integrations – if required	Testing of the bi-directional data exchange and traceability between LIMS and Waters Empower software. This test script would only be applicable to LIMS implementations that include analytical instruments that utilize Empower software.
		LIMS Label Configuration	Verification that all LIMS labels can be printed and that the label content meets user requirements. This test script is typically performed for each label type that has been defined for the system.
		Label Barcode Scan Data Accuracy	Testing that all barcodes can be scanned and verifying that the barcode information is correct and accurate.
		Verification of Barcode Scan Functionality to Add Materials, Supplies, and Equipment.	Testing that scanning of barcodes adds the required information into the system and that any pre-configured actions take place upon barcode scan.
		Verification of Functionality to Select Result Specifications Based on Test Method	Verifying that LIMS correctly applies the result specification based on the selected test method, testing that users can add/remove result specifications as applicable, and verifying that LIMS displays the correct result specification information in the system.
		Verification of Functionality that Records Start and End Date and Time for Instrument Data Collection Steps	Testing of instrument-related steps that automatically or manually date/time metadata to ensure accuracy and data integrity.
		Instrument Maintenance and Calibration Status Notifications and Warnings	Verification that LIMS appropriately flags instruments due or overdue for calibration or maintenance, verification that flags/restrictions are removed when calibration or maintenance is performed, and verification that any overrides (if configured) allow for use of the instrumentation when flagged/restricted.
		Reagent/Supply Expiry and Reorder Notifications and Warnings	Verification that LIMS appropriately flags reagents/supplies due to expire or meet re-order thresholds, verification that LIMS restricts use of reagents/supplies that are expired, verification that any overrides (if configured) allow for use of the reagent/supply when flagged/restricted.
		Data Entry and Import/Addition of Supporting Raw Data Verification	Verification that raw data files, attachments, and manual entries are correctly linked, stored, and retrievable within LIMS.
		Report(s) Configuration, Formatting, and Data Integrity	Verification that all system-generated reports work as expected, are formatted correctly, and include accurate and complete data.
		Electronic Records Archive Functionality	Testing the ability to archive data securely and in compliance with the user-specified retention requirements.
		Electronic Records Restoration from Archive	Validation that archived data can be restored and rendered readable without loss or corruption.
		Date/Timestamp Formatting and Application on Forms and Reports	Verification that all forms and reports that require date/timestamps properly display them in the correct date and time format.
		Stability Analytics Functionality – if required	Validation of stability module functions, including data entry, trend analysis, and expiry determination calculations/logic.
		Audit Trail Functionality and Accuracy	Testing to ensure the LIMS captures accurate audit trails of user activity, data changes, and timestamped records. Data integrity of the audit trail is also tested to ensure it cannot be modified or deleted.
		VPN Configuration, Access, and Encryption	Verification that remote access to the LIMS works as intended, is secure, and is encrypted to the user’s required standards.
PQ	Performed on the Qualification (QUAL) environment	Verification of OQ Completion and Acceptability	The OQ documentation is reviewed to confirm that all LIMS Operational requirements have been met and that no deviations remain open.
		End-to-End Sample Lifecycle Execution	Verification that users with the appropriate roles can log, receive, test, review, and release a sample using approved procedures in LIMS.
		User Role-Based Functionality	Verification that users who are assigned to different job roles (e.g., analyst, reviewer, approver) can perform their expected LIMS functions without unauthorized access.
		Routine Batch Lifecycle Execution – if required	Verification that users with the appropriate roles can initiate, prepare, complete, review, and release a batch using approved procedures in LIMS.
		Stability Study Execution – if required	Verification that users with the appropriate roles can initiate, manage, perform pulls, complete, review, and release a stability study using approved procedures in LIMS.
		Environmental Monitoring Workflow – if required	Verification that users with the appropriate roles can initiate, collect, receive, test, review, and release environmental monitoring samples. This test script would also verify if sampling plans created the required samples at the defined time and that any notifications (if configured) worked as intended.
		System Backup and Restore Verification	Execution of a full system backup and restore to verify that critical data and operations are preserved and accessible after restoration.
		Electronic Record Review and Approval	Verification that electronic records can be reviewed, annotated, and approved according to approved SOPs and are compliant with 21CFR Part 11 requirements.
		Business Continuity Testing – Optional	Perform a simulated (e.g., LIMS outage or data loss event) and verify that business-critical processes can be recovered or completed using the approved backup or alternative workflows.
PQ	Performed on the Production (PROD) environment	LIMS System Analytics Monitoring	Perform 30 days of monitoring of the LIMS Production database and applications servers using software monitors. Typical metrics that are monitored include but are not limited to operational status, CPU and memory utilization, latency, throughput/bandwidth, and input/output operations per second.
		LIMS Monitoring from User Perspective – optional	Evaluation of LIMS performance from a user perspective based on the collection of user observations and service/help requests. This can be concurrent with LIMS System Analytics Monitoring.

^*a*^ Test scripts are subject to change based on system use case and design.

## IOPQS—WHAT, WHEN, AND HOW?

The 21 CFR Part 211 and the FDA Guidance for Industry do not provide details on what to include in the IOPQ, which equipment requires an IOPQ, nor the frequency for requalification studies beyond the initial IOPQ. Instead, these critical parameters are determined by the end user. Industry best practices have shifted to a risk-based approach in accordance with the FDA Quality Systems Approach to Pharmaceutical Current Good Manufacturing Practice Regulations (10). Here, we provide a framework that may be used to design a risk-based equipment qualification program.

Conceptually, the risk assessment is similar to an Individualized Quality Control Plan (IQCP) used in clinical labs to justify alternative options for quality control (often applied to reduce quality control testing frequency and/or scope) (17). The risk assessment for IOPQ development begins with equipment classification to determine whether qualification is needed. This is followed by a secondary risk assessment to determine qualification requirements and frequency for requalification. Equipment classification is based on measurement capability and equipment complexity and can be grouped into three categories as defined in USP <1058 > (18) for qualification and calibration purposes:

- **Group A** includes equipment with the least complexity that does not have, or is used without, measurement capabilities (e.g., stir plates and vortex mixers).
- **Group B** includes equipment that provides measurement capability or maintains conditions that can affect a measurement (e.g., pH meters and pipettes). It may also include equipment that does not have measurement capability but is of moderate complexity (e.g., centrifuges, controlled temperature units, and biosafety cabinets).
- **Group C** includes equipment that has high complexity or a significant degree of computerization (e.g., blood culture instrument, MALDI-TOF MS, document management system, and laboratory information management system). This equipment typically offers some form of measurement capability and is often used to provide a test result.

Based on equipment classification, decisions can be made regarding calibration frequency and whether equipment qualification is required. For example, Group A equipment may not require qualification because it lacks complexity and measurement capability. Groups B and C equipment may require qualification due to the increased complexity or measurement capabilities. The justifications behind qualification requirements, frequency, and qualification test scripts should be established in a Validation Master Plan (VMP) and should be applied consistently to each piece of equipment in the laboratory.

After equipment classification, equipment that has been flagged as requiring qualification can be assessed to determine the level of qualification needed and the qualification frequency (Table 6). A modified Failure Mode and Effects Analysis (FMEA) model is recommended because it provides end-user flexibility. The FMEA model should categorize severity, occurrence, detection, and equipment use frequency (see [19] for details on how to conduct a FMEA). Evaluation begins with the identification of each potential failure mode that is scored 1-5 (Fig. S1a) based on the equipment use case. Scoring should be conducted with a cross-functional team (e.g., engineers, end-users, and quality) to ensure comprehensive and knowledgeable subject matter analysis. After each failure mode is evaluated, the score from each category is multiplied together to calculate the Risk Priority Number (RPN) for the failure mode. The RPN indicates the relative risk associated with each failure mode and can help prioritize which failure modes are more critical. Final risk level determination (low, medium, and high) can be made using the average RPN mapped to a risk priority matrix (Fig. S1b) to establish final qualification requirements for that equipment. At this stage, there is flexibility in determining these requirements as long as they are consistent (based on the VMP) and comply with regulations.

**TABLE 6:** Examples of equipment that may be used in clinical laboratories for cGMP sterility testing, outlining suggested minimum qualification frequency requirements based on risk level^*a*^

Equipment type	Equipment risk level		
	High risk	Medium risk	Low risk
Standalone Electronic Systems (e.g., LIMS, Document Management System)	Initial qualification. No standard requalification window. Use the change control process to assess risk and requalification requirements when major updates occur and/or new functionalities are added.	NA	NA
Blood Culture Systems used for Sterility Testing	Initial qualification and annual calibration	NA	NA
Biosafety Cabinets used for Product Handling	Initial qualification and recertification every 6 months	NA	NA
Centrifuges used to Process Product	Initial qualification and annual calibration	NA	NA
CTUs used for Product Storage or Test Reagent Storage	Requalification biennially and annual calibration	Requalification every4 years and annual calibration	Initial qualification and annual calibration
Manual Pipettes	NA	NA	Calibration only
Microscopes used for Product Gram stain	NA	NA	Initial qualification and routine preventative maintenance
Vortex	NA	NA	Calibration of measurement functions (e.g., time, speed), if relevant. If no measurement functions are on the vortex, no qualification or calibration is needed.

^*a*^ “NA” indicates not applicable.

IOPQ efforts should be placed on the attributes/parameters/functions of equipment that pose the most risk to the operational process. Risk assessment should be conducted when the equipment is first acquired and onboarded, any time changes are proposed to the equipment, as new data are acquired on equipment performance, and when there are changes to the organization’s risk profile.

## DOES THE FDA REALLY REVIEW THE EQUIPMENT IOPQ?

In fiscal years 2023 and 2024 (October 1, 2022, to September 30, 2024), the FDA conducted 35,193 inspections, of which 11,642 (33%) were related to biologics, drugs, and devices (Table S1). Observations made by the FDA during a facility inspection are documented on FDA Form 483. A review of these observations showed that of the 3,264 citations issued for drugs and biologics, 555 (17%) were related to equipment (under 21 CFR 211 and 212; Table S2). For devices (e.g., IVDs [20]), managed under 21 CFR 820, equipment violations accounted for 180 [4.5%] citations out of 4,007 observations (Table S3).

The equipment citations in Tables S2 and S3 mostly relate to the lack of procedures, lack of documentation, and lack of validation. Documentation and data integrity during validation and calibration activities are critical and align with the golden rule of “if it wasn’t documented, it didn’t happen.” In this context, cGMP testing laboratories are subject to the same standards as manufacturing facilities (6). These citations indicate that the level of detail expected by the FDA may be more than the current practices that are generally accepted in clinical labs to satisfy equipment validation (as listed in CAP checklist items COM.30550 and COM.30575).

## IS THE CLINICAL LAB SUBJECT TO EQUIPMENT IOPQ REQUIREMENTS IF ONLY PERFORMING MICROBIAL IDENTIFICATION?

This review has covered the equipment IOPQ requirements that are expected for laboratories performing direct primary testing in support of cGMP activities (e.g., sterility testing and/or environmental monitoring culture incubation). However, in some circumstances, primary culture and incubation may be conducted by the manufacturer themselves, with positive cultures referred to clinical microbiology for microbial identification only. Is the clinical microbiology lab still subject to cGMP requirements if only performing indirect secondary testing, such as microbial identification?

The short answer is yes, although there are caveats. Any laboratory performing work in support of cGMP activities is subject to cGMP requirements, including equipment IOPQ. The FDA expects that qualified vendors that meet cGMP requirements are used for any process within the cGMP framework, including testing, material suppliers, equipment services, etc. If a clinical lab provides indirect testing support (such as microbial identification of a positive culture), the caveat lies in whether a cGMP decision is being made by the manufacturer, which may lead to a cGMP investigation. For example, a manufacturer may make a go/no-go decision regarding product release pending identification from a positive sterility culture. Similarly, a cGMP decision or action may be taken in response to an identification from an in-process environmental culture or gloved-fingertip test. In these circumstances, the clinical lab is open to the risk of inspection. Unfortunately, under current law, demonstration of compliance with CLIA and CAP requirements is negligible because clinical practices and cGMP activities fall under different federal regulatory acts and expectations (Table 1).

## CONCLUSIONS

IOPQs form a comprehensive approach to validating equipment, ensuring that it is suitable for its intended purpose and capable of consistently producing quality results. Clinical laboratories tend to focus most often on PQ through assay verification or validation. The IQ and OQ portions are often overlooked, not performed, and/or not documented, although Phase II CAP requirements exist (COM.30550 and COM.30575). Fortunately, IOPQ does not need to be developed from scratch. Equipment validation protocol templates are common in the pharmaceutical industry and can be made available by vendors to conduct in-house testing or outsourced to a third party. IOPQ, however, is expensive and time-consuming. For example, in our experience, IOPQ for the BacT/ALERT instrument and software conducted by a third-party vendor cost over $20,000 and took over one week to execute, followed by several weeks before a final report was approved (this timeline included vendor report issuance, end-user review, vendor corrections to paperwork, and final end-user quality assurance approval). More recently, we chose to conduct the IOPQ for the BioFire 2.0 Industry System Mycoplasma FilmArray Panel in-house. Using a vendor-provided template, this testing took over 80 h to complete due to our initial inexperience with performing direct IOPQ testing in-house.

In summary, cGMP testing is not only about the analytical test itself. It encompasses a wide range of quality aspects that, from the outset, may look similar to CAP clinical requirements but differ in practice, oversight, and documentation. Laboratory compliance with the applicable regulations (CLIA for clinical, FD&C Act for cGMP) is important. Given current workforce concerns and the changing regulatory landscape, the hesitancy expressed by clinical microbiology laboratory directors in bringing on cGMP testing activities is valid. These concerns, however, should be balanced with the recognition that outsourcing testing to cGMP laboratories may contribute to slower delivery of therapy, which may be negatively impacting patient care (personal communication concerns raised at the AABB Annual Meeting 2024, Houston, TX, and in discussion with cellular therapy lab directors in academic settings). A risk-based balance must be reached while still adhering to regulatory requirements. To achieve this, a working group that includes members of the ASM clinical microbiology community and the AABB cellular therapy community has begun conversations with a goal of developing, with FDA input, practical guidance and clarification on the level of cGxP practices required for sterility testing of Section 361 products. Furthermore, the information provided here is relevant if challenges to the regulatory scope of LDTs (i.e., falling back under the FD&C Act instead of CLIA) and classification of LDTs as “devices” were to occur again in the future.

## SUPPLEMENTAL MATERIAL

Figures S1a and S1b jcm.01477-24-s0001.docx Risk score and matrix.

Tables S1, S2, and S3 jcm.01477-24-s0002.docx FDA inspection findings for equipment violations.

ASM does not own the copyrights to Supplemental Material that may be linked to, or accessed through, an article. The authors have granted ASM a non-exclusive, world-wide license to publish the Supplemental Material files. Please contact the corresponding author directly for reuse.

## References

[1] United States Food and Drug Administration. 2025. Approved cellular and gene therapy products, on united states food and drug administration. Available from: https://www.fda.gov/vaccines-blood-biologics/cellular-gene-therapy-products/approved-cellular-and-gene-therapy-products

[2] Saez-Ibañez AR, Upadhaya S, Partridge T, Winkelman D, Correa D, Campbell J. 2024. The changing landscape of cancer cell therapies: clinical trials and real-world data. Nat Rev Drug Discov 23:736–737. doi:10.1038/d41573-024-00094-4 38822118

[3] Cundell T, Atkins JW, Lau AF. 2023. Sterility testing for hematopoietic stem cells. J Clin Microbiol 61:e0165422. doi:10.1128/jcm.01654-22 36847535 PMC10035301

[4] Gebo JET, East AD, Lau AF. 2021. A side-by-side comparison of clinical versus current good manufacturing practices (cgmp) microbiology laboratory requirements for sterility testing of cellular and gene therapy products. Clin Microbiol Newsl 43:181–191. doi:10.1016/j.clinmicnews.2021.10.001

[5] Gebo JET, Lau AF. 2020. Sterility testing for cellular therapies: what is the role of the clinical microbiology laboratory? J Clin Microbiol 58. doi:10.1128/JCM.01492-19 PMC7315024 32321785

[6] United States Food and Drug Administration. 2004. Guidance for industry sterile drug products products by aseptic processing - current good manufacturing practice. United States Food and Drug Administration, White Oak, MD.

[7] Parenteral Drug Association. 2016. PDA technical report no.56 (TR 56): application of phase-appropriate quality system and cGMP to the development of therapeutic protein drug substance (API or biological active substance). Parenteral Drug Association, Bethesda, MD.

[8] DeMarco E. 2015. Contamination scare at NIH leaves clinical trial subjects with tough choice. American Association for the Advancement of Science, Washington DC.

[9] United States Food and Drug Administration. 2025. Facts about the current good manufacturing practice (CGMP), on United States food and drug administration. Available from: https://www.fda.gov/drugs/pharmaceutical-quality-resources/facts-about-current-good-manufacturing-practice-cgmp

[10] United States Food and Drug Administration. 2006. Guidance for industry quality systems approach to pharmaceutical CGMP regulations. United States Food and Drug Administration.

[11] United States District Judge (Sean. D. Jordan). 2025. American clinical laboratory association et al v. U.S. food and drug administration et al (civil no. 4:24-CV-479-SDJ); association for molecular pathology et al v. U.S. food and drug administration et al (civil no. 4:24-CV-824-SDJ). United States Court Eastern District of Texas Sherman Division

[12] Aaron DG, Adashi EY, Cohen IG. 2024. The US FDA’s new rule for regulating laboratory-developed tests. JAMA Health Forum 5:e242917. doi:10.1001/jamahealthforum.2024.2917 39392638

[13] Miller MB, Watts ML, Samuel L. 2024. FDA’s proposed rule for the regulation of laboratory-developed tests. J Clin Microbiol 62:e0148823. doi:10.1128/jcm.01488-23 38206042 PMC10865810

[14] The Pew Charitable Trusts. 2021. The role of lab-developed tests in the in vitro diagnostics market. Available from: https://www.pewtrusts.org/en/research-and-analysis/reports/2021/10/the-role-of-lab-developed-tests-in-the-in-vitro-diagnostics-market#:~:text=In%202014%2C%20FDA%20estimated%20that,develop%20such%20tests%20did%20so.&text=CLIA%20standards%20differ%20from%20those%20applied%20during%20FDA%20premarket%20review

[15] United States Food and Drug Administration. 2011. Guidance for industry process validation: general principles and practices. United States Food and Drug Administration, Silver Spring, MD.

[16] United States Pharmacopeia. 2022. USP <1223> validation of alternative microbiological methods. Rockville, MD United States Pharmacopeia

[17] United States Centers for Medicare & Medicaid Services. 2025. Individualized quality control plan (IQCP), on United States centers for medicare & medicaid services. Available from: https://www.cms.gov/medicare/quality/clinical-laboratory-improvement-amendments/quality-control

[18] United States Pharmacopeia. 2017. USP <1058> analytical instrument qualification. Rockville, MD United States Pharmacopeia

[19] American Society for Quality. 2025. FMEA. Available from: https://asq.org/quality-resources/fmea

[20] United States Food and Drug Administration. 2024. Overview of IVD regulation, on United States food and drug administration. Available from: https://www.fda.gov/medical-devices/ivd-regulatory-assistance/overview-ivd-regulation

