# Supplementary material for: All you need to know about equipment validation for sterility testing
Source: J Clin Microbiol. 2025 Aug 11;63(9):e01477-24. doi: 10.1128/jcm.01477-24 (PMC12421869; doi:10.1128/jcm.01477-24)
Supplement: Figures S1a and S1b — Risk score and matrix. [file jcm.01477-24-s0001.docx]

Supplemental Figure 1a: Example Factor Risk Scores Used for Equipment FMEA.

| **Factor**  **Risk Score** | **Factor Risk Score Meanings** | | | |
| --- | --- | --- | --- | --- |
|  | **Likelihood of Failure** | **Impact of Failure** | **Detectability of Failure** | **Use Frequency** |
| **5** | Certain, 100% | Failure impacts product and/or testing rendering them unusable or out of compliance. | Very Unlikely, < 5% | Used in every process. |
| **4** | High Likelihood | Failure impacts product and/or testing rendering tests invalid and unrepeatable or impacts product in such a manner that end users deem it is can be used at risk. | Low Likelihood | Used more often than not. |
| **3** | Likely, 50% | Failure impacts product and/or testing rendering tests invalid but repeatable or impacts product in such a way that it can be remediated by the end user. | Likely, 50% | Used just as much as it is not used. |
| **2** | Low Likelihood | Minor impact to product and/or testing that is within acceptable limits. Testing is not rendered invalid, or product is not detrimentally harmed. | High Likelihood | Used less often than not. |
| **1** | Very Unlikely, < 5% | No impact to product and/or testing. | Certain, 100% | Very rarely used. |

Supplemental Figure 1b: Example Risk Priority Number Matrix used for equipment FMEA. Green are Low Risk, Yellow are Medium Risk, and Red are High Risk.

|  |  | **Likelihood of Failure** | | | | |  |  |
| --- | --- | --- | --- | --- | --- | --- | --- | --- |
|  |  | **1** | **2** | **3** | **4** | **5** |  |  |
| **Impact of Failure** | **1** | 1 | 4 | 9 | 16 | 25 | **1** | **Use Frequency** |
|  | **2** | 4 | 16 | 36 | 64 | 100 | **2** |  |
|  | **3** | 9 | 36 | 81 | 144 | 225 | **3** |  |
|  | **4** | 16 | 64 | 144 | 256 | 400 | **4** |  |
|  | **5** | 25 | 100 | 225 | 400 | 625 | **5** |  |
|  |  | **1** | **2** | **3** | **4** | **5** |  |  |
|  |  | **Detectability of Failure** | | | | |  |  |
