# Supplementary material for: All you need to know about equipment validation for sterility testing
Source: J Clin Microbiol. 2025 Aug 11;63(9):e01477-24. doi: 10.1128/jcm.01477-24 (PMC12421869; doi:10.1128/jcm.01477-24)
Supplement: Tables S1, S2, and S3 — FDA inspection findings for equipment violations. [file jcm.01477-24-s0002.docx]

**Supplemental Table 1.** Total number of inspections conducted by the FDA in fiscal years (FY) 2023 and 2024.

| **Product Type** | **FY 2023**  Oct 1, 2022, to Sep 30, 2023 | **FY 2024**  Oct 1, 2023, to Sep 30, 2024 | **Grand Total** |
| --- | --- | --- | --- |
| Biologics | 2,151 | 1,460 | 3,611 |
| Devices | 2,405 | 2,100 | 4,505 |
| Drugs | 1,750 | 1,776 | 3,526 |
| Food/Cosmetics | 10,902 | 10,132 | 21,034 |
| Tobacco | 122 | 130 | 252 |
| Veterinary | 1,209 | 1,056 | 2,265 |
| **Grand Total** | 18,539 | 16,654 | 35,193 |

**Supplemental Table 2.** Summary of FDA citations related to equipment for drugs and biologics in FY23 and FY24 (n=555).

| **Reference Number** | **Short Description(s)** | **Example Long Description(s)** | **Frequency** | **% of Total Citations (3,264)** |
| --- | --- | --- | --- | --- |
| 21 CFR 211.68(b) | Backup data not assured as exact and complete | Backup data is not assured as exact, complete and secure from alteration, erasure or loss through keeping hard copy or alternate systems. Electronic signatures based on are used, but they do not meet the requirements of 21 CFR Part 11. | 107 | 3.3% |
|  | Backup file not maintained | Failure to maintain a backup file of data entered into the computer or related system. |  |  |
|  | Computer control of master formula records | Appropriate controls are not exercised over computers or related systems to assure that changes in master production and control records or other records are instituted only by authorized personnel. Electronic records are used, but they do not meet audit trail and employee accountability/responsibility policy requirements to ensure that they are trustworthy, reliable and generally equivalent to paper records. |  |  |
|  | Input/output verification | Input to and output from the computer, related systems of formulas and records or data are not checked for accuracy. |  |  |
|  | Written record not kept of program and validation data | A written record of the program along with appropriate validation data has not been maintained in situations where backup data is eliminated by computerization or other automated processes. |  |  |
| 21 CFR 211.63 | Equipment design, size, and location | Equipment used in the manufacture, processing, packing or holding of drug products is not of appropriate design, of adequate size and suitably located to facilitate operations for its intended use and cleaning and maintenance. | 99 | 3.0% |
| 21 CFR 211.67(a) | Cleaning / Sanitizing / Maintenance | Equipment and utensils are not cleaned, maintained and sanitized at appropriate intervals to prevent malfunctions and contamination that would alter the safety, identity, strength, quality or purity of the drug product. | 93 | 2.8% |
| 21 CFR 211.67(b) | Written procedures fail to include | Written procedures for cleaning and maintenance fail to include description in sufficient detail of methods, equipment and materials used, description in sufficient detail of the methods of disassembling and reassembling equipment as necessary to assure proper cleaning and maintenance and parameters relevant to the operation. | 72 | 2.2% |
|  | Written procedures not established/followed | Written procedures are not established and followed for the cleaning and maintenance of equipment, including utensils, used in the manufacture, processing, packing or holding of a drug product. |  |  |
| 21 CFR 211.68(a) | Calibration/Inspection/Checking not done | Routine calibration, inspection and checking of automatic, mechanical and electronic equipment is not performed according to a written program designed to assure proper performance. | 53 | 1.6% |
|  | Written calibration/inspection records not kept | Records of the calibration checks and inspections of automatic, mechanical or electronic equipment, including computers or related systems are not maintained. |  |  |
| 21 CFR 211.160(b)(4) | Establishment of calibration procedures | Procedures describing the calibration of instruments, apparatus, gauges and recording devices are not written or followed and deficiently written or followed. | 32 | 1.0% |
|  | Instruments, apparatus, et. al. not meeting specs | The use of instruments, apparatus and recording devices not meeting established specifications was observed. |  |  |
|  | Test devices not meeting specifications | Test devices are deficient in that instruments and recording devices not meeting established specifications are used. |  |  |
|  | Written calibration procedures | Written calibration procedures for instruments, apparatus, gauges, and recording devices are deficient in that they do not include specific directions, schedules, limits for accuracy and precision and provisions for remedial action if limits are not met. |  |  |
|  | Calibration – at intervals, written program, remedial action | The calibration of instruments, apparatus and gauges is not done at suitable intervals in accordance with an established written program and with provisions for remedial action in the event accuracy and/or precision limits are not met. |  |  |
| 21 CFR 211.182 | Chronological order of equipment log entries | The entries in the equipment cleaning and use logs are not in chronological order. | 19 | 0.6% |
|  | Personnel dating/signing equipment log | The persons performing and double-checking the cleaning and maintenance are not signing or initialing the equipment cleaning and use log. |  |  |
|  | Specific information required in individual logs | Individual equipment logs do not show time, date, product and lot number of each batch processed. |  |  |
|  | Written records kept in individual logs | Written records of major equipment cleaning, maintenance and use are not included in individual equipment logs. |  |  |
| 21 CFR 211.67(b)(3) | Cleaning SOPs/instructions | Procedures for the cleaning and maintenance of equipment are deficient regarding sufficient detail of the methods, equipment, and materials used in the cleaning and maintenance operation, and the methods of disassembly and reassembling equipment as necessary to assure proper cleaning and maintenance. | 18 | 0.6% |
| 21 CFR 211.46(b) | Equipment for environmental control | Equipment for adequate control over air pressure, micro-organisms, dust, humidity and temperature is not provided when appropriate for the manufacture, processing, packing or holding of a drug product. | 11 | 0.3% |
| 21 CFR 211.113(b) | Validation lacking for sterile drug products | Procedures designed to prevent microbiological contamination of drug products purporting to be sterile did not include adequate validation of the aseptic and sterilization process. | 10 | 0.3% |
| 21 CFR 606.60(a) | Equipment observed, standardized, calibrated | Equipment used in the collection, processing and storage and distribution of blood and blood components is not observed, standardized and calibrated on a regularly scheduled basis as prescribed in the SOP Manual. | 9 | 0.3% |
|  | Maintain and clean equipment | Failure to maintain equipment used in the collection, processing, storage and distribution of blood and blood products. |  |  |
|  | Provide proper equipment to meet requirements | Failure of equipment to perform in the manner for which it was designed so as to assure compliance with the official requirements prescribed in 21 CFR 606. |  |  |
| 21 CFR 211.67(c) | Cleaning/maintenance records not kept | Records are not kept for the maintenance, cleaning, sanitizing and inspection of equipment. | 6 | 0.2% |
| 21 CFR 211.67(b)(6) | Cleaning SOPs/inspection | Procedures for the cleaning and maintenance of equipment are deficient regarding inspection of the equipment for cleanliness immediately before use. | 6 | 0.2% |
| 21 CFR 211.42(c)(10)(vi) | Equipment to control conditions | Aseptic processing areas are deficient regarding systems for maintaining any equipment used to control the aseptic conditions. | 4 | 0.1% |
| 21 CFR 212.30(b) | Equipment procedures | [Firm] did not implement procedures and document [Firm] activities in accordance with [Firm] procedures to ensure that all equipment is cleaned that could reasonably be expected to adversely affect the identity, strength, quality, or purity of a PET drug, or give erroneous or invalid test results when improperly used or maintained. | 4 | 0.1% |
| 21 CFR 211.67(b)(5) | Cleaning SOPs/equipment protection | Procedures for the cleaning and maintenance of equipment are deficient regarding the protection of clean equipment from contamination prior to use. | 3 | 0.1% |
| 21 CFR 211.194(d) | Laboratory equipment calibration records | Laboratory records do not include complete records of the periodic calibration of laboratory instruments and apparatus. | 3 | 0.1% |
| 21 CFR 606.60(b) | Equipment calibration frequency | Equipment used in the collection, processing and storage and distribution of blood and blood components is not observed, standardized and calibrated with at least the frequency required. | 2 | 0.1% |
| 21 CFR 211.65(a) | Equipment construction – reactive surfaces | Equipment surfaces that contact components are reactive, additive or absorptive so as to alter the safety, identity, strength, quality, or purity of the drug product beyond the official or other established requirements. | 1 | < 0.1% |
| 21 CFR 211.67(b)(2) | Cleaning SOPs/schedules | Procedures for the cleaning and maintenance of equipment are deficient regarding maintenance and cleaning schedules, including, where appropriate, sanitizing schedules. | 1 | < 0.1% |
| 21 CFR 212.30(c) | Contact surfaces | [Firm’s] equipment is not constructed so that surfaces that contact components, in-process materials and PET drugs are not reactive, additive, or absorptive so as to alter the quality of the PET drugs. | 1 | < 0.1% |
| 21 CFR 212.60(e) | Equipment | All equipment used to perform the testing is not suitable for its intended purposes and capable of producing valid results. | 1 | < 0.1% |

**Supplemental Table 3.** Summary of FDA citations related to equipment for devices in FY23 and FY24 (n=180).

| **Reference Number** | **Short Description(s)** | **Example Long Description(s)** | **Frequency** | **% of Total Citations (4,007)** |
| --- | --- | --- | --- | --- |
| 21 CFR 820.72(a) | Calibration, Inspection, etc. Procedures Lack of or Inadequate | Procedures to ensure equipment is routinely calibrated, inspected, checked and maintained have not been adequately established. | 88 | 2.2% |
|  | Equipment control activity documentation | Equipment calibrations, inspections, checks and maintenance activities have not been documented. |  |  |
|  | Equipment suitability & capability | Certain inspection, measuring and test equipment is not suitable for its intended purposes and capable of producing valid results. |  |  |
| 21 CFR 820.70(i) | Documentation of software validation | Software validation activities and results for computers or automated data processing systems used as part of the quality system have not been adequately documented. | 38 | 1.0% |
|  | Software validation for automated processes | Software used as part of production has not been validated for its intended use according to an established protocol. Electronic records are used, but they do not meet systems validation, system access limitation and audit trail requirements to ensure that they are trustworthy, reliable and generally equivalent to paper records. |  |  |
|  | Validation of changes to automated process software | Changes to software used as part of the quality system were not adequately validated before approval and issuance. |  |  |
| 21 CFR8 20.70(g)(1) | Maintenance schedule, Lack of or inadequate schedule | Schedules for the adjustment, cleaning, and other maintenance of equipment have not been adequately established. | 22 | 0.5% |
| 21 CFR 820.72(b) | Calibration procedures - content | Calibration procedures do not include specific directions and limits for accuracy and precision. | 20 | 0.5% |
|  | Remedial action | When test/measurement equipment was found to not meet accuracy and precision limits, no action was taken to evaluate whether there was any adverse effect on the device's quality. |  |  |
|  | Remedial action - documentation | Evaluations of out-of-calibration equipment and remedial actions taken were not documented. |  |  |
| 21 CFR 820.70(g) | Equipment Installation, Placement, Specified Requirements | The appropriate design, construction, placement, and installation of manufacturing equipment have not been ensured. | 6 | 0.1% |
| 21 CFR8 20.70(g)(2) | Periodic equipment inspection lack of or inadequate procedure | Procedures for conducting periodic inspections to ensure adherence to equipment maintenance schedules have not been adequately established. | 6 | 0.1% |
|  | Periodic equipment inspections | Periodic inspections of equipment were not conducted to ensure adherence to applicable maintenance schedules. |  |  |
